# The Relation between Serum Uric Acid and HbA1c Is Dependent upon Hyperinsulinemia in Patients with Newly Diagnosed Type 2 Diabetes Mellitus

**DOI:** 10.1155/2016/7184123

**Published:** 2016-06-14

**Authors:** Yuliang Cui, Hemei Bu, Xianghua Ma, Sha Zhao, Xiaona Li, Shan Lu

**Affiliations:** Department of Endocrinology, The First Affiliated Hospital of Nanjing Medical University, Nanjing 210029, China

## Abstract

*Objective*. The aim of our study was to explore the dependent condition of the relationship between uric acid and blood glucose in type 2 diabetes.* Research Design and Methods*. We measured the HbA1c, serum uric acid, creatinine, lipids profiles, and so forth of 605 newly diagnosed type 2 diabetes patients, and oral glucose tolerance tests (OGTTs) were performed on each patient. The population was divided into high and low insulin groups. Multiple linear regression analyses were conducted to assess the relationship between uric acid and HbA1c.* Results*. Serum uric acid and HbA1c levels were low in newly diagnosed type 2 diabetes patients. However, we found no significant relationship between uric acid and HbA1c by regression analysis after adjusting total insulin. The concentration of uric acid was inversely correlated with HbA1c in the high insulin group, regardless of patient sex. However, no associations were found in low insulin group.* Conclusions*. The negative correlation between uric acid and HbA1c is conditional in newly diagnosed type 2 diabetes patients and is related to hyperinsulinemia. Therefore, uric acid is likely only useful as a biomarker of blood glucose in patients exhibiting hyperinsulinemia.

## 1. Introduction

Type 2 diabetes mellitus is one of the most common and disabling metabolic diseases; its increasing incidence has become a serious threat to human health [1]. Increasing evidence suggests that hyperuricemia is an independent risk factor for impaired fasting glucose (IFG) and type 2 diabetes. Patients with hyperuricemia are at a significantly higher risk of progressing to type 2 diabetes [2, 3]. A large number of researchers have begun to consider uric acid as a serum indicator of glycometabolic disorders, because of a correlation between uric acid and glucose metabolism [4, 5]. However, changes in serum uric acid and blood glucose do not exhibit a linear relationship, as some have assumed. Rather, the relationship follows more of a bell curve. Uric acid levels rise with increasing blood glucose concentrations in the normal and prediabetes population. However, in type 2 diabetes patients, uric acid levels tend to decline with increasing blood glucose concentrations [6, 7].

The reasons for the inverse relationship between uric acid and blood glucose in type 2 diabetes mellitus remain unclear. However, insulin levels are also closely related to uric acid levels. Serum uric acid levels will increase with increasing serum insulin levels in diabetic patients [8, 9]. Whether insulin is the factor that influences the relationship between the uric acid and blood glucose is not clear. Previous studies did not provide an explanation. Thus, our study aimed to further explore the connection between uric acid and blood glucose in newly diagnosed type 2 diabetes.

## 2. Research Design and Methods

### 2.1. Study Population

We performed a retrospective analysis on the inpatient database in the First Affiliated Hospital of Nanjing Medical University (China) between 2008 and 2014. We chose 605 individuals (435 males and 170 females) aged 30–74-years with newly diagnosed type 2 diabetes. The diagnosis for type 2 diabetes was in accordance with the diagnostic criteria promulgated by the World Health Organization (WHO) in 1990. Screening criteria were as follows: (1) patients must have newly diagnosed type 2 diabetes and have never received hypoglycemic drugs, diet, or exercise therapy prior to admission to the hospital. (2) Patients must have never received any drugs that would affect blood glucose, insulin, or serum uric acid before testing. (3) Patients must have no history of serious liver or kidney problems or infection, trauma, or stress. All patients were admitted to our hospital to screen for diabetic complications and to evaluate islet-beta cell function after they were diagnosed with type 2 diabetes (FBG≧7.0 or PBG≧11.1) in the outpatient department. There was no stress hyperglycemia or transient hyperglycemia caused by other reasons. There were 800 cases that were evaluated. Among them, 58 patients were excluded because they had taken drugs affecting uric acid, while 89 cases were excluded because they had impaired liver or kidney functions and 48 were not included because their data were incomplete.

### 2.2. Physical Examination and Laboratory Methods

We analyzed the age, history of diabetes, height, weight, systolic blood pressure (SBP), and diastolic blood pressure (DBP) in all the patients. Venous blood samples were collected after fasting for more than 8 hours. The serology indexes, such as triglyceride (TG), creatinine (Cr), and serum uric acid (SUA) levels, were measured by automatic biochemical analyzer. Glycated hemoglobin (HbA1c) was measured by high-performance liquid chromatography (HPLC). The oral glucose tolerance test (OGTT) and insulin release test (IRT) were performed in all patients. Blood samples were collected at fasting (0 min) and at 30, 60, and 120 minutes after taking 75 g anhydrous glucose to measure plasma glucose (dextrose oxidase method) and serum insulin (radioimmunoassay).

### 2.3. Calculations

The body mass index (BMI) was calculated by dividing the weight (kg) by the height (m) squared. Total insulin level was represented as the area under curve of insulin during 0–120 min of the IRT [10], which was obtained from the irregular trapezoid method (Area = 1/2∑_*i*=1_^*n*^ *X*_*i*−1_(*Y*_*i*−1_ + *Y*_*i*_)) [11].

### 2.4. Statistical Analysis

All statistical analyses were computed using SPSS for Windows, version 17.0. Measurement data were expressed as $\bar{x}\pm s$. All of the comparisons between measurement data in line with the normal distribution were analyzed by independent samples *t*-test. A test of normality was performed for all the arguments, and the variables showing skewed distribution that took natural logarithms were converted to meet the normal distribution. Pearson correlation tests and multivariate linear regression analyses were used to assess the relationship between HbA1c and InsAUC120, the relationship between serum uric acid and InsAUC120, and the relationship between serum uric acid and HbA1c. The data were divided according to the two types of patient groups: a low insulin group and a high insulin group, based on the median InsAUC120 level in male versus female gender groups [10]. The correction between serum uric acid and HbA1c was evaluated within each group. A *P* value of <0.05 (two-tailed) was considered to be statistically significant.

## 3. Results

**Figure 1:**
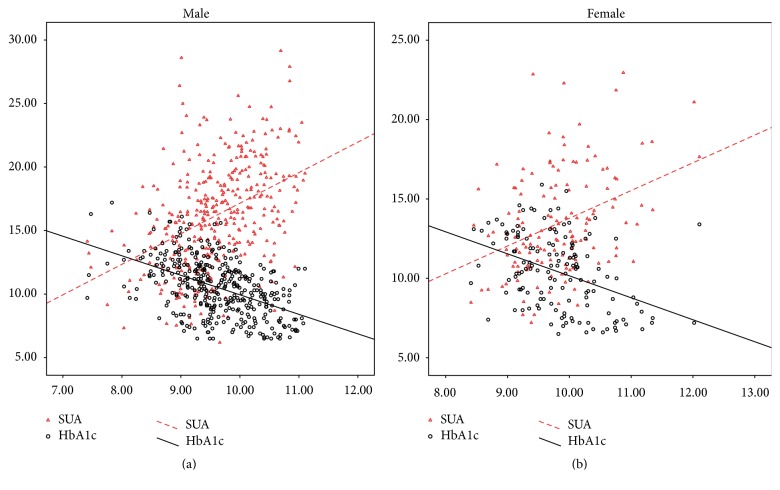
Scatter plots of the correlation between InsAUC120 and SUA as well as HbA1c in different genders. The abscissa is the area under the curve of insulin (InsAUC120). Both uric acid and HbA1c change with the increase in InsAUC120. However, the trends of the two correlation curves are opposite, in either male or female. Serum uric acid is directly correlated with insulin level, and HbA1c is inversely associated with insulin level.

In Table 1, the general clinical data gathered for each gender group is presented. Regardless of gender, HbA1c was inversely correlated with InsAUC120 (Figure 1, Table 2). The correlation still existed after adjusting possible influencing factors, such as age, BMI, SBP, DBP, TG, creatinine, and SUA by multivariate regression analysis (Table 3). Serum uric acid was positively correlated with InsAUC120 (Figure 1, Table 2). The correlation still existed after adjusting possible influencing factors, such as age, BMI, SBP, DBP, HbA1c, TG, and creatinine by multivariate regression analysis (Table 3). Serum uric acid showed a negative association with HbA1c in each gender group (male: *r* = −0.224, *P* = 0.000; female: *r* = −0.245, *P* = 0.000). When HbA1c increased by one unit, the serum uric acid decreased by 3.868 units in male group and by 6.036 units in female group after adjusting age, BMI, SBP, DBP, TG, and creatinine (Table 3). Interestingly, after adding InsAUC120 to the regression equation, the relationship between uric acid and HbA1c no longer existed (Table 3). For both males and females, serum uric acid was inversely correlated with HbA1c levels in high insulin group (male: *r* = −0.281, *P* = 0.015; female: *r* = −0.331, *P* = 0.006). The relationship still existed after adjusting possible influencing factors such as age, BMI, SBP, DBP, TG, and creatinine by multivariate regression analysis (Table 4). However, no correlation between SUA and HbA1c was present in the low insulin group (male: *r* = −0.102, *P* = 0.087; female: *r* = −0.047, *P* = 0.704; Table 4).

## 4. Discussion

Uric acid is a final product of purine catabolism. It has been confirmed that elevated uric acid levels can increase the risk of metabolic syndrome, atherosclerosis, and chronic kidney disease [12–14]. Over the years, the association between uric acid levels and glucose metabolism has been a hot research topic. A growing number of studies have indicated that there is a “bell” fit between uric acid and glucose concentrations. Uric acid levels tend to decrease after their first increase, along with an increase in blood glucose concentration [15–17]. A number of studies have found that serum uric acid levels are inversely correlated with blood glucose concentrations in type 2 diabetes patients. However, until now, it has been unclear as to why this relationship exists and what factors influence this relationship.

Previous studies have been carried out mainly in patients with preexisting type 2 diabetes. In such patients, the levels of uric acid and blood glucose are easily influenced by alimentary control or medication. For such studies, a single blood sugar reading was taken, which does not represent the total behavior of fluctuating blood sugar levels susceptible to interference/control by medical intervention. In order to explore the relationship between uric acid and blood glucose more accurately, we chose patients with newly diagnosed type 2 diabetes (at time of diagnosis immediately prior to medical intervention) to eliminate the influence of drugs or dietary control. In addition, we used glycosylated hemoglobin (HbA1c) as a measure of blood glucose metabolism, not affected by occasional fluctuations in blood sugar. Our study revealed that, among both the male and female patients with newly diagnosed type 2 diabetes, uric acid levels showed a significant upward trend, while the HbA1c levels were significantly lower and correlated with an increase in InsAUC120 levels. This data suggested that a change in insulin level may lead to a degree of correlation between uric acid and HbA1c. Subsequently, we found that the serum uric acid levels were inversely correlated with HbA1c levels, regardless of patient gender. However, the correlation between uric acid and HbA1c disappeared after adjusting InsAUC120 through multiple linear regression analysis. This further indicated that the correlation between uric acid and HbA1c may be affected by InsAUC120. Therefore, the correlation between uric acid and HbA1c was analyzed by stratified analysis according to insulin level. Only in the case of high insulin levels, we found that serum uric acid was inversely correlated with HbA1c. There was no association between uric acid and HbA1c in patients with low insulin levels. Therefore, the correlation between uric acid and HbA1c most likely relies on insulin levels. Modan et al. [10] showed that there were differences in uric acid concentration among the general population, those with impaired glucose tolerance, and newly diagnosed type 2 diabetes patients, only when hyperinsulinemia existed. However, if patients had normal insulin levels, there were no differences in serum uric acid levels, regardless of blood glucose levels. Therefore, the findings of Modan et al. agree with our results. The reasons for the above situation may be due to the effect of insulin on the metabolism of uric acid and glucose. Hyperinsulinemia could increase the activation of the hexose phosphate shunt, which would promote the biosynthesis and transformation of purine, thus increasing the rate of uricogenesis [18]. At the same time, insulin may increase reabsorption of uric acid from the kidneys by stimulating the urate anion transporter on the border membrane in the proximal tubular brush [19], the end result of which is an increase in the concentration of serum uric acid. High insulin levels naturally lead to lower blood glucose concentrations. Therefore, insulin may control the concentration of uric acid and blood glucose at the same time, explaining the inverse correlation between uric acid and blood glucose when blood insulin levels are high.

In conclusion, our study confirmed that there is an inverse correlation between uric acid and HbA1c, which is dependent on hyperinsulinemia in patients with newly diagnosed type 2 diabetes. High insulin levels may be an important factor affecting the correlation between the uric acid and HbA1c. Out results suggest that uric acid might serve as a biomarker of blood glucose, but only under conditions of hyperinsulinemia.

## Figures and Tables

**Table 1:** Basic/clinical characteristics of study participants by gender and the difference between males and females ($\bar{x}\pm s$).

General indexes	Male (*n* = 435)	Female (*n* = 170)	*t*	*P*
Age (yr)	51.22 ± 12.65	56.14 ± 9.83	−4.561	0.000^*∗*^
BMI (kg/m^2^)	25.91 ± 3.56	25.18 ± 3.45	2.262	0.024^*∗*^
SBP (mmHg)	134.40 ± 18.09	131.70 ± 15.71	1.813	0.071
DBP (mmHg)	84.19 ± 10.93	82.13 ± 9.56	2.280	0.023^*∗*^
HbA1c (%)	10.45 ± 2.24	10.40 ± 2.36	0.257	0.797
ALT (U/L)	35.66 ± 28.80	31.93 ± 27.30	1.453	0.147
AST (U/L)	27.23 ± 18.35	28.45 ± 21.67	−0.696	0.478
TC (mmol/L)	4.99 ± 1.68	5.25 ± 1.10	−1.837	0.067
TG (mmol/L)	2.39 ± 1.51	1.60 ± 0.85	3.397	0.001^*∗*^
HDL (mmol/L)	1.10 ± 0.40	1.26 ± 0.75	−4.117	0.000^*∗*^
LDL (mmol/L)	3.57 ± 0.82	3.39 ± 0.34	0.252	0.801
BUN (mmol/L)	5.68 ± 2.82	5.01 ± 1.36	2.932	0.003^*∗*^
Cr (*μ*mol/L)	73.56 ± 15.93	54.44 ± 10.04	17.624	0.000^*∗*^
SUA (*μ*mol/L)	325.70 ± 84.28	272.65 ± 66.74	8.133	0.005^*∗*^

SBP: systolic pressure, DBP: diastolic pressure, HbA1c: glycosylated hemoglobin, ALT: glutamic-pyruvic transaminase, AST: glutamic oxaloacetic transaminase, TC: total cholesterol, TG: triglycerides, HDL: high density lipoprotein, LDL: low density lipoprotein, BUN: blood urea nitrogen, Cr: creatinine, and SUA: serum uric acid. The *t*-test with different samples was adopted for comparison between groups. ^*∗*^ *P* < 0.05 was considered as statistically significant difference.

**Table 2:** Correlations between InsAUC120 and SUA as well as HbA1c in different genders.

	Male		Female	
	*r*	*P*	*r*	*P*
HbA1c	−0.483	0.000^*∗*^	−0.456	0.000^*∗*^
SUA	0.406	0.000^*∗*^	0.368	0.000^*∗*^

InsAUC120: the area under the curve of insulin from 0 min to 120 min. Serum uric acid is positively related to insulin level and HbA1c is negatively associated with insulin level in both male and female patients. ^*∗*^ *P* < 0.05 denotes statistical significance.

**Table 3:** Multiple linear regression for SUA, HbA1c, and InsAUC120.

	Male				Female			
	*B*	*β*	*t*	*P*	*B*	*β*	*t*	*P*
Model 1: the dependent variable is HbA1c								
Age (yr)	0.007	0.038	0.662	0.508	0.061	0.265	3.198	0.002^*∗*^
BMI (kg/m^2^)	0.028	0.045	0.752	0.452	0.133	0.190	2.302	0.023^*∗*^
SBP (mmHg)	−0.003	−0.027	−0.474	0.636	0.009	0.060	0.692	0.490
DBP (mmHg)	−0.012	−0.057	−1.01	0.314	−0.007	−0.038	−0.444	0.658
TG (mmol/L)	0.009	0.006	0.112	0.911	1.091	0.143	1.772	0.079
Cr (*μ*mol/L)	−0.014	−0.082	−1.524	0.128	−0.027	−0.114	−1.429	0.156
SUA (*μ*mol/L)	−0.001	−0.041	−0.710	0.478	−0.004	−0.109	−1.324	0.188
InsAUC120	−1.434	−0.456	−7.864	0.000^*∗*^	−1.573	−0.464	−5.465	0.000^*∗*^

Model 2: the dependent variable is SUA								
Age (yr)	−1.753	−0.267	−5.470	0.000^*∗*^	0.152	0.022	0.262	0.793
BMI (kg/m^2^)	5.708	0.241	5.044	0.000^*∗*^	1.178	0.055	0.690	0.491
SBP (mmHg)	0.544	0.116	2.288	0.023^*∗*^	0.219	0.049	0.556	0.579
DBP (mmHg)	−0.515	−0.066	−1.311	0.190	0.058	0.010	0.110	0.912
HbA1c (%)	−3.868	−0.103	−2.355	0.019^*∗*^	−6.036	−0.209	−2.617	0.010^*∗*^
TG (mmol/L)	4.914	0.088	1.972	0.049^*∗*^	50.428	0.234	2.953	0.004^*∗*^
Cr (*μ*mol/L)	2.043	0.310	6.913	0.000^*∗*^	0.891	0.127	1.583	0.115

Model 3: the dependent variable is SUA								
Age (yr)	−1.694	−0.257	−4.945	0.000^*∗*^	0.723	0.111	1.167	0.245
BMI (kg/m^2^)	3.136	0.135	2.419	0.016^*∗*^	0.608	0.031	0.329	0.743
SBP (mmHg)	0.592	0.131	2.434	0.015^*∗*^	0.105	0.025	0.260	0.795
DBP (mmHg)	−0.439	−0.057	−1.06	0.288	0.061	0.011	0.115	0.908
HbA1c (%)	−1.369	−0.037	−0.710	0.478	−3.751	−0.132	−1.324	0.188
TG (mmol/L)	2.839	0.051	1.049	0.295	21.665	0.100	1.117	0.266
Cr (*μ*mol/L)	1.787	0.277	5.643	0.000^*∗*^	0.835	0.126	1.421	0.158
InsAUC120	26.771	0.230	3.924	0.000^*∗*^	19.767	0.206	1.997	0.038^*∗*^

SBP: systolic pressure, DBP: diastolic pressure, HbA1c: glycosylated hemoglobin, TG: triglycerides, Cr: creatinine, SUA: serum uric acid, and InsAUC120: the area under the curve of insulin from 0 to 120 min. Both uric acid and HbA1c have a regressive relationship with InsAUC120 and some related influencing factors, regardless of gender. There is no regressive relationship between SUA and HbA1c after adjusting InsAUC120. ^*∗*^ *P* < 0.05 denotes statistical significance.

**Table 4:** Multiple linear regression for SUA and HbA1c in patients with high and low insulin levels.

	Male				Female			
	*B*	*β*	*t*	*P*	*B*	*β*	*t*	*P*
High insulin levels: the dependent variable is SUA								
Age (yr)	−2.062	−0.355	−4.346	0.000^*∗*^	1.224	0.181	1.324	0.191
BMI (kg/m^2^)	4.480	0.200	2.644	0.009^*∗*^	3.382	0.149	1.207	0.232
SBP (mmHg)	0.156	0.033	0.383	0.702	0.714	0.170	0.976	0.333
DBP (mmHg)	0.164	0.023	0.263	0.793	−0.601	−0.083	−0.474	0.637
HbA1c (%)	−4.923	−0.111	−1.582	0.016^*∗*^	−9.995	−0.329	−2.450	0.007^*∗*^
TG (mmol/L)	5.363	0.099	1.382	0.169	4.654	0.049	0.382	0.704
Cr (*μ*mol/L)	1.670	0.244	3.262	0.001^*∗*^	0.124	0.018	0.136	0.892

Low insulin levels: the dependent variable is SUA								
Age (yr)	−0.973	−0.143	−1.858	0.065	0.577	0.097	0.631	0.531
BMI (kg/m^2^)	3.017	0.118	1.561	0.121	−0.138	−0.008	−0.055	0.956
SBP (mmHg)	0.792	0.201	2.520	0.013	−0.338	−0.086	−0.585	0.561
DBP (mmHg)	−1.276	−0.166	−2.152	0.033	0.345	0.078	0.587	0.560
HbA1c (%)	−2.850	−0.062	−1.202	0.131	0.230	0.008	0.056	0.956
TG (mmol/L)	0.372	0.007	0.096	0.924	12.483	0.156	1.108	0.273
Cr (*μ*mol/L)	1.974	0.352	4.782	0.000^*∗*^	1.400	0.226	1.723	0.090

Serum uric acid has an independent relationship with HbA1c through multiple stepwise regression analysis in patients with hyperinsulinemia, while there is no association between SUA and HbA1c in patients with hypoinsulinemia. ^*∗*^ *P* < 0.05 denotes statistical significance.
